# Rapid Lymphatic Dissemination of Encapsulated Group A Streptococci *via* Lymphatic Vessel Endothelial Receptor-1 Interaction

**DOI:** 10.1371/journal.ppat.1005137

**Published:** 2015-09-09

**Authors:** Nicola N. Lynskey, Suneale Banerji, Louise A. Johnson, Kayla A. Holder, Mark Reglinski, Peter A. C. Wing, David Rigby, David G. Jackson, Shiranee Sriskandan

**Affiliations:** 1 Faculty of Medicine, Imperial College London, London, United Kingdom; 2 MRC Human Immunology Unit, Weatherall Institute of Molecular Medicine, University of Oxford, Oxford, United Kingdom; The University of Alabama at Birmingham, UNITED STATES

## Abstract

The host lymphatic network represents an important conduit for pathogen dissemination. Indeed, the lethal human pathogen group A streptococcus has a predilection to induce pathology in the lymphatic system and draining lymph nodes, however the underlying basis and subsequent consequences for disease outcome are currently unknown. Here we report that the hyaluronan capsule of group A streptococci is a crucial virulence determinant for lymphatic tropism *in vivo*, and further, we identify the lymphatic vessel endothelial receptor-1 as the critical host receptor for capsular hyaluronan in the lymphatic system. Interference with this interaction *in vivo* impeded bacterial dissemination to local draining lymph nodes and, in the case of a hyper-encapsulated M18 strain, redirected streptococcal entry into the blood circulation, suggesting a pivotal role in the manifestation of streptococcal infections. Our results reveal a novel function for bacterial capsular polysaccharide in directing lymphatic tropism, with potential implications for disease pathology.

## Author Summary

Pathogens are known to invade the host not only via the systemic circulation but also via the lymphatic network, however the mechanisms underlying the latter route and the consequences for disease outcome have not been well studied. The important human pathogen, group A streptococcus, is responsible for a number of clinical syndromes affecting both the lymphatic vessels and draining lymph nodes, such as lymphangitis and lymphadenitis. How such pathologies are orchestrated, and their significance in the development of serious infection are currently unknown. In this study, we show that the hyaluronan capsule secreted by group A streptococcus is critical for bacterial spread to draining lymph nodes, and we demonstrate that this occurs as a result of a specific interaction with the lymphatic vessel endothelial receptor-1. Genetic deletion or functional blockade of this receptor prevented streptococcal transit to draining lymph nodes in a murine model of infection, which in turn enhanced bacterial spread into the blood circulation. Together these results define a novel interaction between the group A streptococcal capsule and the lymphatic endothelial receptor-1 as a critical axis in the establishment of lymphatic tropism for this pathogen, with clear implications for disease severity in the host.

## Introduction

Lymphatic dissemination of intracellular bacteria and viruses is a well characterized mechanism of pathogenic invasion in the host, which occurs independently of transit through blood [1–3]. In contrast, exploitation of lymphatics by extracellular bacterial pathogens has received scant attention, despite clear clinical evidence that such pathogens can induce pathology within the lymphatic system [4,5].

Group A streptococcus (GAS) is one such important, exclusively human, extracellular pathogen. Pathology in the host is initiated by breach of mucosal surfaces and subsequent tissue destruction, resulting in a diverse disease spectrum spanning the superficial (pharyngitis, pyoderma) to the systemic (necrotizing fasciitis, toxic shock syndrome) as well as subsequent post-infection immune sequelae (rheumatic fever) [6]. Combined, these manifestations of GAS disease account for the 4^th^ highest mortality rate amongst bacterial pathogens [7].

The onset of invasive bacterial disease is dependent on the appropriately timed expression of an arsenal of virulence factors which facilitate pathogen dissemination within the host. The group A streptococci produce over 40 virulence factors, of which the hyaluronan (HA; (GlcNAc_β1–4_-GlcUA)_n_) capsule [8] expressed by almost all serotypes is critical in defense against neutrophil-mediated opsonophagocytosis [9]. Elaboration of this capsule further contributes to GAS pathogenesis by facilitating colonization of the host through adhesive interactions between HA and its well characterized receptor, CD44, in pharyngeal epithelium [10,11]. This interaction results in bacterial translocation and subsequent invasion of host tissue *via* the paracellular route [12]. We and others have previously shown that GAS have a particular ability to disseminate from a focus of infection to locally draining lymph nodes [13,14], a feature that, experimentally, has been associated with systemic dissemination. Clinically, GAS has been associated with lymphatic pathologies such as lymphadenitis and lymphangitis [4,5]. At present however, the host factors underlying this apparent lymph tropism and lymphoid tissue dissemination are completely unknown.

In addition to CD44, a number of other HA binding proteins are expressed in the human host [15,16]. Lymphatic vessel endothelial receptor (LYVE)-1 is a member of the HA binding Link protein superfamily that bears 41% amino acid sequence similarity to CD44 [16]. LYVE-1 is almost exclusively expressed in lymph vessels and lymph node sinuses, a system where CD44 is completely absent, and has been implicated in binding of HA from interstitial matrix and in HA-mediated leukocyte trafficking [16, 17]. However, the propensity for HA-producing pathogens to hijack this pathway as a means of lymphatic tropism has not been explored.

In this work, we provide the first evidence to implicate the HA capsule as a critical bacterial determinant of GAS lymphoid tropism. We further demonstrate that a specific adhesive interaction between capsular HA and LYVE-1 is pivotal for GAS dissemination to draining lymph nodes *in vivo*, thus defining a novel conduit for GAS translocation, with potentially important implications for the establishment of systemic disease.

## Results

### Capsular HA is a critical factor for lymphatic trafficking of GAS *in vivo*

**Fig 1:**
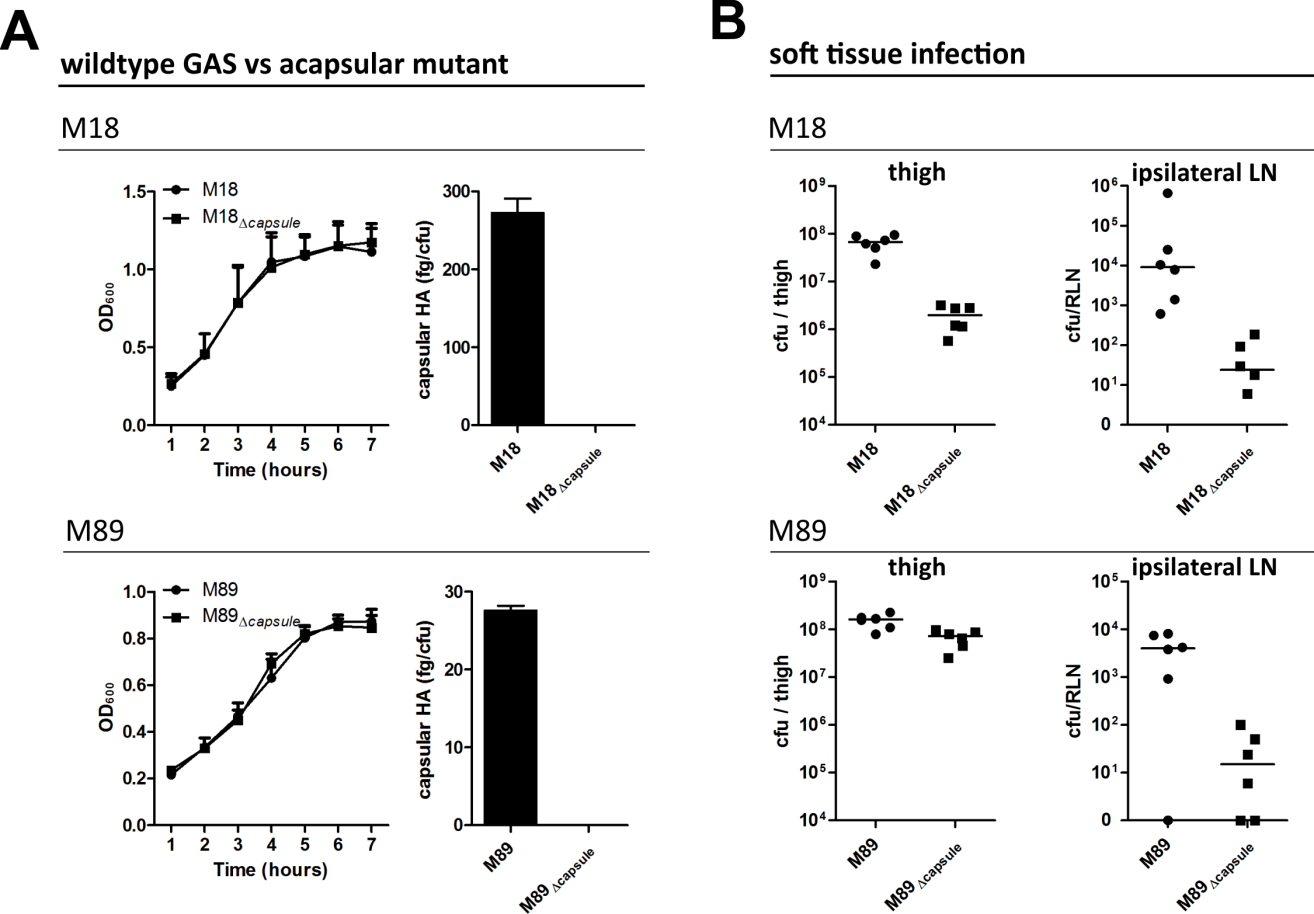
Capsular HA is critical for GAS dissemination to lymphatics. **(**A) Generation of isogenic acapsular strains of M18 and M89 GAS. Left-right; Comparison of bacterial growth by optical density (600 nm) and quantification of capsular HA expression expressed as fg of HA per colony forming unit. (B) Dissemination of wildtype and acapsular GAS in murine soft-tissue infection. Left to right; Bacterial burden (cfu) in ipsilateral lymph node and thigh 3 hours post-injection (n = 6 mice/group; line depicts median).

The streptococcal virulence factors which govern bacterial transit to lymph nodes remain poorly understood. We hypothesized that the HA capsule could play an important role in this tropism, due to both its anti-phagocytic and adhesive properties. Since the degree of encapsulation varies greatly between GAS serotypes [8], and capsular HA is known to occlude other surface expressed adhesins [18,19], we generated isogenic hyaluronan synthase (*hasA*) mutant strains in two important serotypes; the hyper-encapsulated GAS serotype M18, and the lower density capsule serotype M89, to allow for unambiguous assessment of capsule involvement (Fig 1A). Importantly, the total ablation of HA capsule expression had no discernible effect on *in vitro* bacterial growth rate in either GAS strain (Fig 1A).

Soft tissue infections represent an important GAS disease manifestation, which can readily be modelled in mice. Such models allow reproducible measurement of spread to locally draining lymph nodes at early time points following thigh muscle infection, prior to development of any severe signs of sepsis. Accordingly, we utilized a murine thigh muscle infection model to investigate the role of capsular HA in bacterial lymphatic tropism. Significantly, expression of capsular HA was clearly associated with enhanced bacterial transit to draining lymph nodes *in vivo*. Three hours after intra-muscular infection, the bacterial burden in the draining ipsilateral inguinal lymph node was markedly greater in mice infected with wildtype encapsulated GAS than that observed for mice infected with isogenic acapsular mutant GAS (Fig 1B). This was true for both the M18 and M89 isogenic strain pairs. Importantly, there was virtually no spread to the contralateral node, indicating that bacterial transit to draining lymph node was *via* afferent lymphatics and not blood circulation.

Loss of capsule expression is known to increase sensitivity of GAS to opsonophagocytic clearance by neutrophils in a serotype dependent manner, a phenomenon to which M18 strains are particularly sensitive [9]. Consistent with this, we detected a 1.5 log-fold reduction in bacterial burden at the site of infection in mice infected with isogenic acapsular M18_Δcapsule_ compared with wildtype M18 (Fig 1B), which corresponded with a significant increase in neutrophil-mediated uptake of bacterial cocci in an *in vitro* opsonophagocytosis assay (S1 Fig). Despite the observed differences in clearance from muscle between M18 and M18_Δcapsule_, an almost three log-fold difference was observed between these isogenic strains upon dissemination to the draining ipsilateral lymph node (Fig 1B). In the case of serotype M89, loss of capsule had barely any effect on bacterial clearance from muscle, consistent with *in vitro* opsonophagocytosis data (S1 Fig). Similar to serotype M18 however, this loss of capsule led to a 3-log fold reduction in transit to the draining inguinal lymph node. These results provide direct evidence of a new and previously unanticipated role for the GAS HA capsule in directing lymph tropism and lymphatic spread.

### Capsular HA interacts directly with human LYVE-1

Having established a link between HA capsule expression and the capacity of GAS to disseminate to draining lymph nodes *via* the lymphatics (Fig 1), we considered the possibility that the process was mediated by interaction with LYVE-1, the major HA receptor expressed in human and murine lymphatic vessels, which do not express CD44 [16,20]. We have previously reported that endogenous LYVE-1 displays only residual levels of constitutive HA binding, both *in vitro* and *in vivo*, due in part to heavy modification with auto-inhibitory sialylated O-linked glycans [21]. Hence, in preliminary experiments we performed adhesion assays with recombinant human (hu) and murine (m) LYVE-1 expressed by transfected COS 7 fibroblasts, a cell type with reduced sialyltransferase activity that permits efficient binding of soluble high molecular weight HA. Although GAS showed no ability to bind to untransfected COS 7 cells, encapsulated GAS bound both species of LYVE-1 transfectant avidly, and in a manner that was specifically inhibited by LYVE-1 blocking mAb (S2 Fig).

**Fig 2:**
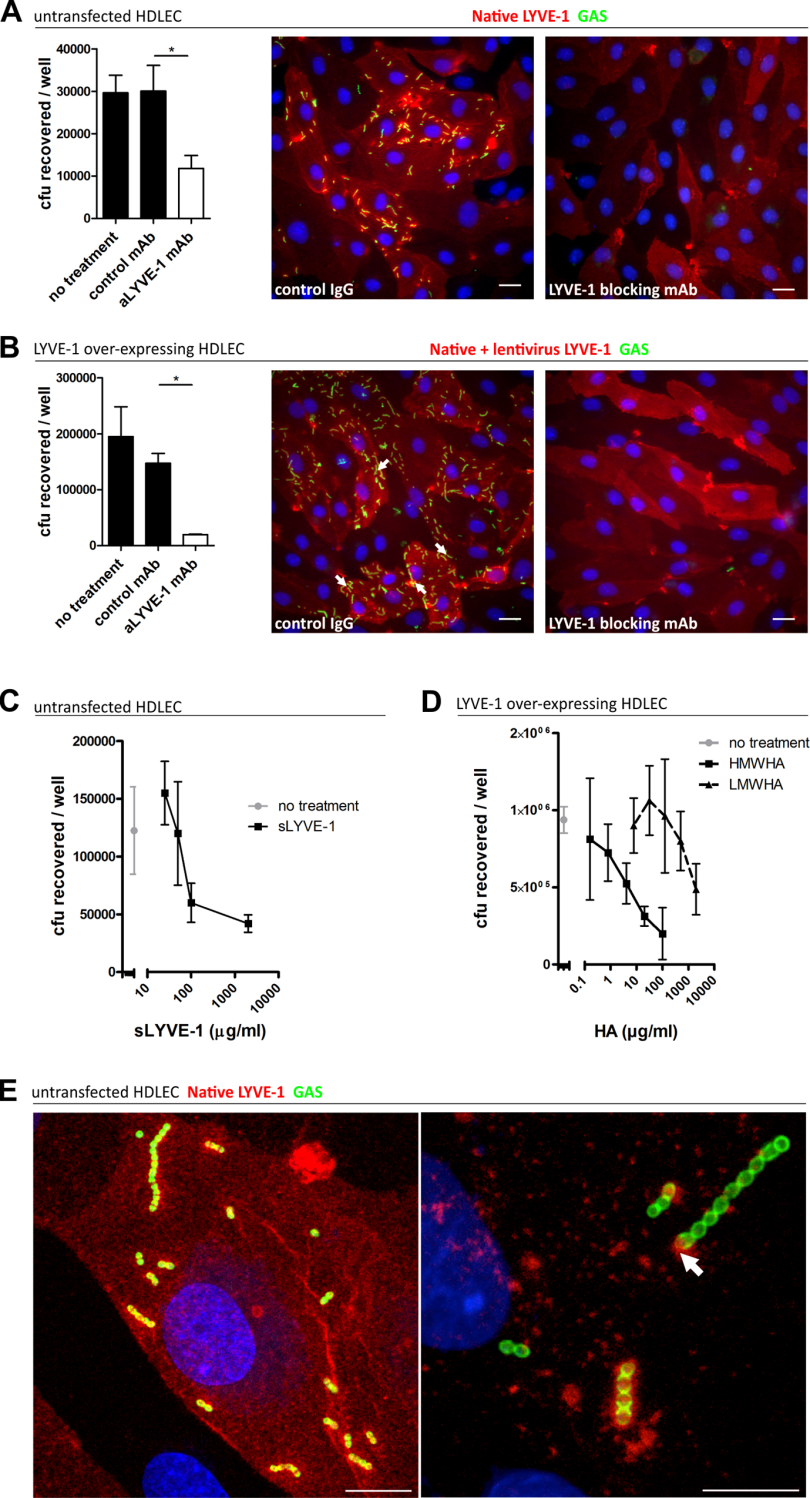
Human LYVE-1 mediates GAS adhesion to HDLEC *via* capsular HA. A-D: Adhesion of encapsulated M18 GAS untransfected and LYVE-1 lentivirus-transfected HDLECs as indicated above each panel. (A and B) Left to right; numbers of adherent GAS were determined by quantitative culture and representative fluorescence microscopy of adherent GAS (30 min incubation) in the presence of control mAb or LYVE-1 blocking mAb. Scale bars (20 μm). (C) Dose-dependent inhibition of GAS adhesion to HDLEC by a soluble human LYVE-1 ectodomain fragment. (D) Dose-dependent inhibition of GAS adhesion to lentivirus transfected HDLECs in the presence of high (HMWHA, solid line) and low (LMWHA, dashed line) molecular weight HA. (A-D: n = 4, data represent mean+/-SD) (Mann Whitney U;* = p<0.05). (E) High resolution confocal microscopy of M18 GAS following infection of HDLEC. Scale bars (10 μm). Red = LYVE-1, green = GAS, blue = nuclei. Arrows indicate encapsulated GAS adhering to clusters of LYVE-1.

To determine whether native sialylated LYVE-1 can support binding of capsular HA we performed quantitative adhesion experiments with monolayers of primary lymphatic endothelial cells isolated from human dermis (HDLEC), which express abundant levels of the receptor on the cell surface [22]. Encapsulated GAS bound efficiently to HDLEC, and the adherent streptococci could be seen to outline the cell borders in a distribution that closely matched that of LYVE-1 (Fig 2A). Moreover, encapsulated GAS also showed increased binding to HDLEC that had been engineered to over-express human LYVE-1 by lentivirus-mediated gene transduction (Fig 2B). Binding to native and lentivirus transfected HDLEC was decreased 3 and 8 fold respectively following incubation with LYVE-1 HA-blocking mAbs (Fig 2A and 2B), and up to 3-fold by competition with a soluble recombinant human LYVE-1 full ectodomain fragment (Fig 2C). Further to this we demonstrated that free high molecular weight HA (>1 x 10^6^ Da) could compete with encapsulated GAS for LYVE-1 binding in a dose-dependent manner, resulting in an almost 5-fold reduction in bacterial adhesion to lentivirus transfected HDLEC, whereas low molecular weight HA (7.5 x 10^3^ Da) could not (Fig 2D). Importantly, addition of a CD44 HA-blocking mAb had no effect on adhesion (S3 Fig). Together these data indicate that the interaction between GAS and the lymphatic endothelium was indeed specific and LYVE-1 mediated.

Intriguingly, higher magnification images of HA encapsulated GAS adhering to the surface of native untransfected HDLEC revealed the individual streptococcal chains to be anchored to variable sized patches of LYVE-1 tethered by single or multiple cocci (Fig 2E). This suggests that the configuration of HA within the bacterial capsule, unlike free high molecular weight HA, can engage the natively sialylated normally quiescent LYVE-1 in lymphatic endothelium through receptor clustering.

### Acapsular GAS do not interact with LYVE-1

The HA capsule constitutes the outermost surface of GAS, and has been demonstrated to mask both fibronectin binding proteins and M-protein [18,19,23]. Indeed, ablation of capsular HA can enhance GAS adhesion to a variety of cell types [19], likely due to the subsequent exposure of addtional bacterial surface proteins, able to interact with host receptors. Consistent with this, isogenic acapsular GAS displayed enhanced adhesion to both native and LYVE-1 supertranfected HDLEC when compared with parental encapsulated strains (S4 Fig), as has been described previously for other primary human cells [11]. In stark contrast to the preference shown by HA encapsulated GAS for adherence at endothelial junctions, acapsular cocci aggregated randomly over the endothelial cell bodies, and in a manner that was wholly independent of LYVE-1 adhesion (S4 Fig). The observed clumping may well result from increased GAS-GAS interaction upon loss of capsule and the interaction with LECs is most likely mediated by integrins as described previously for the analogous interaction of non-encapsulated GAS with epithelial cells [11].

### HA encapsulated GAS bind murine lymphatic endothelium

**Fig 3:**
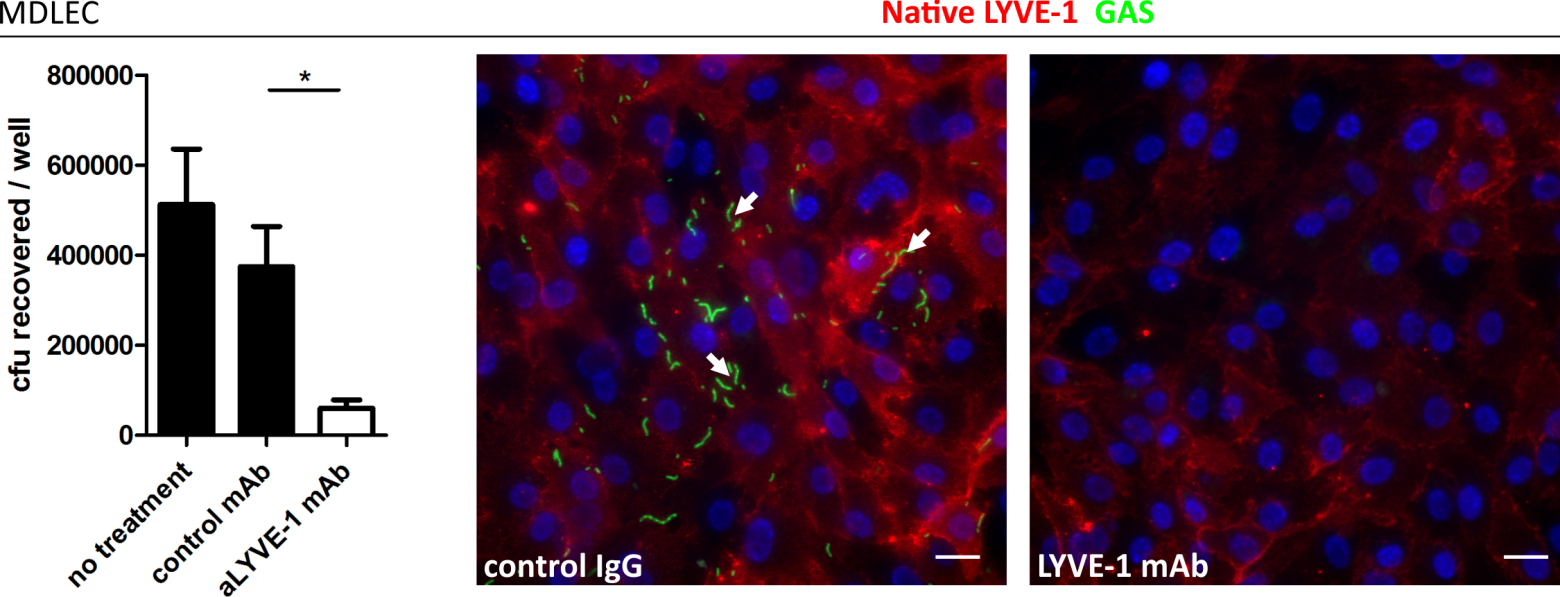
Murine LYVE-1 mediates GAS adhesion to MDLEC *via* capsular HA. Adhesion of M18 GAS to MDLECs. Left to right; numbers of adherent GAS were determined by quantitative culture (n = 4; Data represent mean+/-SD) (Mann Whitney U;* = p<0.05) and representative fluorescence microscopy of adherent GAS (30 min incubation) in the presence of control mAb or LYVE-1 blocking mAb. Scale bars (20 μm). Red = LYVE-1, green = GAS, blue = nuclei. Arrows indicate encapsulated GAS adhering to clusters of LYVE-1.

The structures of human and murine LYVE-1 are very similar, and as such we hypothesized that capsular HA would be sufficient to mediate specific interactions between GAS and native murine LYVE-1. We assessed this by performing adhesion experiments with monolayers of primary lymphatic endothelial cells isolated from murine dermis (MDLEC), which express native LYVE-1 on the cell surface [22]. In common with HDLECs, MDLECs were able to support binding of encapsulated M18 GAS via the LYVE-1 receptor, as demonstrated by inhibition of this interaction by inclusion of a specific murine LYVE-1 HA blocking mAb (Fig 3). Similar to HDLECs, acapsular GAS exhibited clumping and HA-independent adhesion that did not involve LYVE-1 binding. These results, from both human and murine primary cells firmly establish LYVE-1 as a specific lymphatic endothelial adhesion receptor for HA encapsulated GAS *in vitro*.

**Fig 4:**
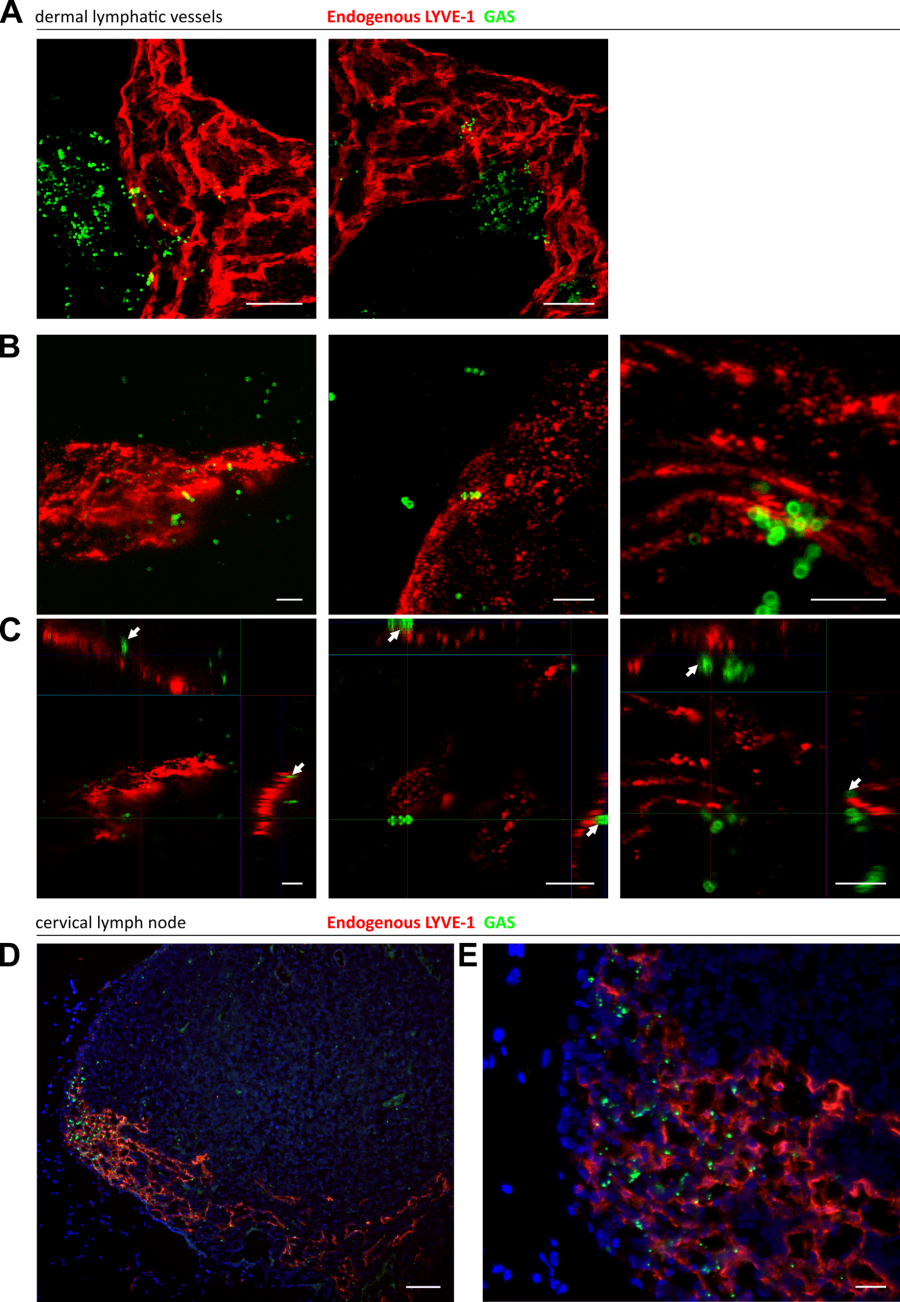
Trafficking of GAS visualized in dermal lymphatic vessels and draining cervical lymph nodes of the mouse ear pinna. A-C: Confocal microscopic images of murine ear skin 6–24 hours after local intradermal inoculation. (A) Low magnification images of lymphatic vessels in skin and surrounding infected tissue 24 hours post infection (scale bar 20 μm). (B) 3D rendered images and (C) orthogonal views of the same z-stacks at 6 hours post infection. Arrows indicate individual streptococci interacting with LYVE-1 dense regions of lymphatic vessel endothelium (scale bar 5 μm). D-E: Epifluorescence microscopic images of frozen sections of draining cervical nodes 6 hours post infection at low power (100X magnification)(D) and high power (400X)(E) (Scale bar 50 μm and 20 μm respectively). Red = LYVE-1, green = GAS, blue = nuclei. Arrows indicate encapsulated GAS adhering to clusters of LYVE-1.

In order to visualize lymph-borne dissemination of GAS *in vivo*, mice were infected intra-dermally in the ear pinna, and confocal microscopy of whole mount sections was utilized to image bacterial translocation through surrounding lymphatic vessels to the cervical lymph node draining the site of infection (Fig 4A–4C). Although few images captured bacteria within the lumen of LYVE-1^+^ dermal lymph vessels, we could nevertheless observe that GAS were concentrated in LYVE-1 rich regions, particularly the paracortical regions that receives afferent lymph from the ear and adjacent dermis (Fig 4D and 4E).

### GAS translocation to draining lymph nodes is impaired in LYVE-1 deficient mice

Having shown that the HA capsule is critical for lymphatic dissemination of GAS in the mouse and identified LYVE-1 as the main adhesion receptor for capsular GAS in primary HDLEC and MDLEC, we next determined whether LYVE-1 plays a role in GAS dissemination *in vivo*.

**Fig 5:**
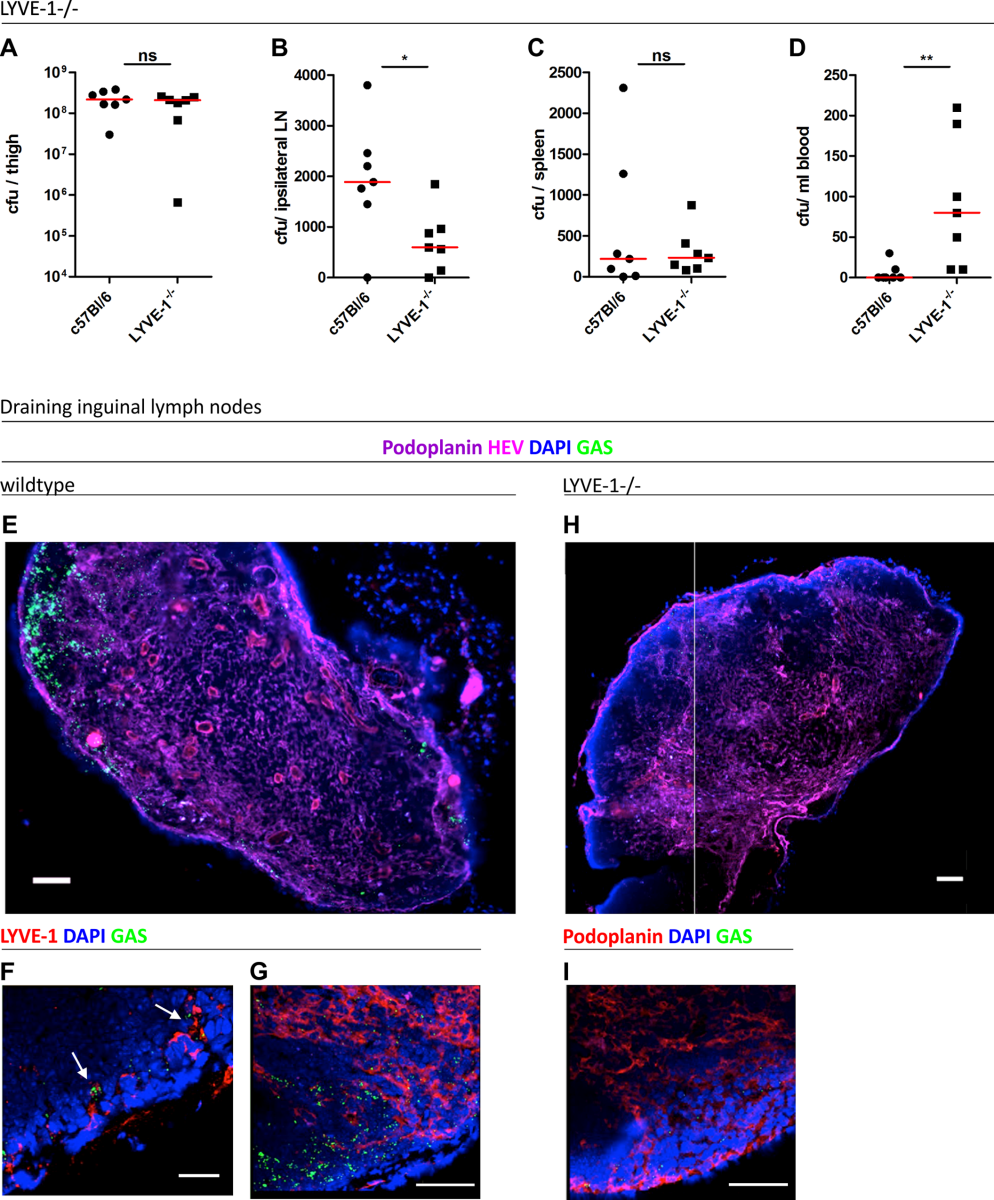
Genetic deletion of LYVE-1 impairs GAS dissemination to draining lymph nodes. Dissemination of M18 GAS in murine soft-tissue infection in constitutive LYVE-1^-/-^ mice (n = 7/group). Numbers of GAS recovered at site of infection (A), ipsilateral draining LN (B), spleen (C) and blood (D) 3 hours post-infection. Lines depict median values in each case (Mann Whitney U;* = p<0.05, ** = p<0.01). (E-I) Confocal micrographs of frozen sections of representative draining inguinal lymph nodes from wildtype (n = 5) (E-G) and LYVE-1^-/-^ (n = 4) (H and I) mice that were resected 3 hours post infection. Original magnification was 100X (E and H) or 400X (F, G and I). Scale bars are 100, 50, 20, 100 and 50 μm for E, F, G, H and I respectively.

Accordingly, to explore the consequences of total LYVE-1 deletion on the course of lymph-borne GAS infection we measured streptococcal transit to draining lymph nodes in constitutive LYVE-1 ^-/-^ mice. As previously reported, these mice have no obvious developmental defects, and have apparently normal lymphatic architecture, fluid clearance and immune function [24]. Following infection *via* an intra-muscular route, the dissemination of encapsulated M18 GAS was compared between LYVE-1^-/-^ mice and age and sex matched wildtype controls by quantitative culture. Importantly no difference was observed in bacterial burden at the site of infection (Fig 5A). However, considerably fewer viable streptococci were recovered from the draining ipsilateral inguinal lymph nodes of LYVE-1^-/-^ mice compared with wildtype mice (Fig 5B), consistent with a functional role for LYVE-1 in streptococcal uptake and / or nodal transit through lymphatic vessels. Interestingly, there was a corresponding rise in the number of bacteria recovered from the blood of LYVE-1^-/-^ animals compared with wildtype (Fig 5C and 5D). This suggests HA-encapsulated M18 GAS can switch to the systemic circulation when access to the lymphatics is impeded.

In order to visualize the location of M18 GAS in the lymph nodes draining the site of infection, the same intra-muscular thigh infection model was used, and inguinal lymph nodes from both wildtype C57Bl/6 and LYVE-1^-/-^ were dissected and sectioned at the 3 hour time-point. Hyper-encapsulated M18 GAS were readily visible in 3/5 of the inguinal lymph nodes resected from infected wildtype mice (Fig 5E–5G), consistent with the results of quantitative culture experiments (Fig 5B). Moreover, while discrete clumps of streptococci could be seen in contact with LYVE-1^+^ sinuses within these nodes, the bacteria were particularly prominent in the paracortical regions (Fig 5G). Importantly, when lymph nodes from LYVE-1^-/-^ mice were imaged for comparison, these in all cases (0/4) contained very few bacteria (Fig 5H) and hardly any were detectable within the paracortical region (Fig 5I). These findings further underline the functional importance of LYVE-1 for lymphatic dissemination of encapsulated M18 GAS and support the conclusion that LYVE-1 may regulate the initial entry of bacteria to the afferent lymphatic vessels.

### Functional blockade of LYVE-1 in mice impairs GAS dissemination to draining lymph nodes

**Fig 6:**
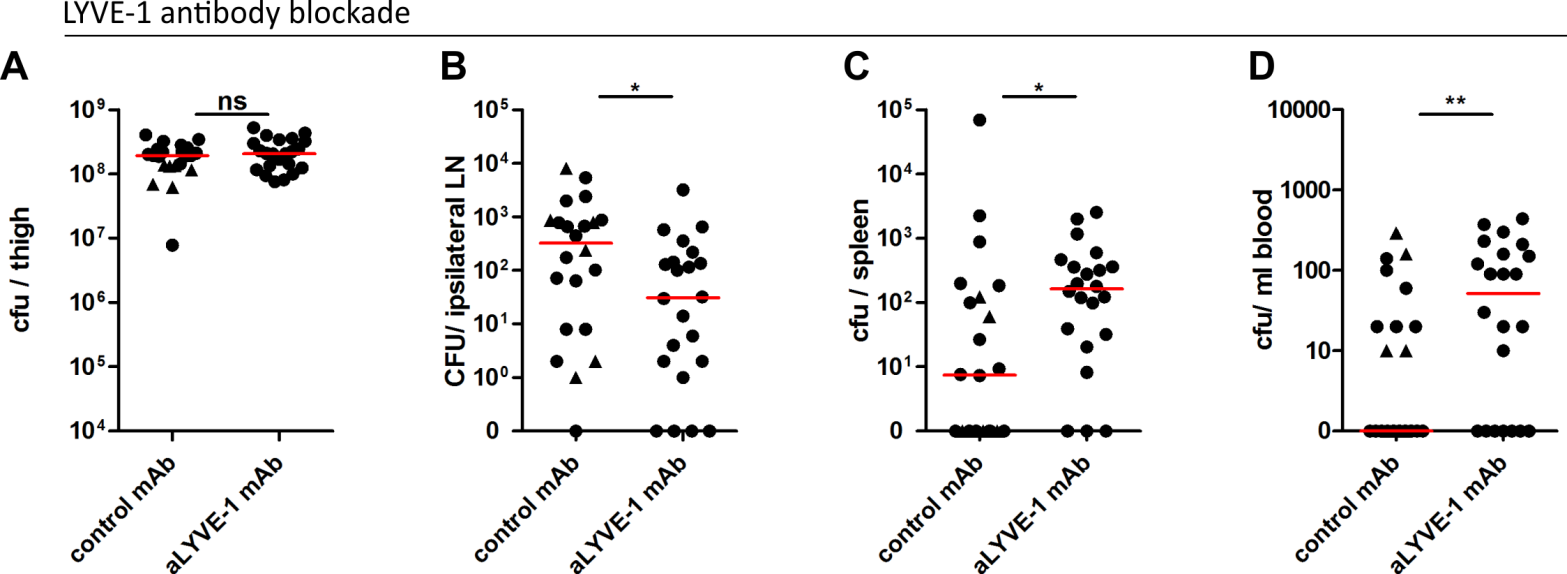
LYVE-1 functional blockade reduces GAS dissemination to draining lymph nodes. Dissemination of M18 GAS in murine soft-tissue infection following LYVE-1 mAb blockade (n = 22/group). Quantitative culture of GAS at site of infection (A), ipsilateral draining LN (B), spleen (C) and blood (D) 3 hours post-infection. Lines depict median values in each case (Mann Whitney U;* = p<0.05, ** = p<0.01). (Control antibody group: circles indicate isotype control antibody and triangles indicate polyclonal control IgG).

Finally, we explored the consequences of LYVE-1 functional blockade in a mouse model of soft tissue infection, with pre-administration of either LYVE-1 HA blocking mAb or control immunoglobulin. Dissemination of M18 GAS to the draining ipsilateral inguinal lymph node and persistence of bacteria within the thigh muscle injection site were quantified three hours post-infection. There was no difference in bacterial burden at the site of infection at this time point (Fig 6A), however quantification of bacteria recovered from the ipsilateral lymph node revealed a ten-fold reduction in GAS dissemination following pre-treatment with LYVE-1 mAb (Fig 6B). A similar result was also observed in mice pre-treated with LYVE-1 mAb following infection with M89 GAS (S5 Fig), confirming the same key role for LYVE-1 in the translocation of encapsulated GAS to draining lymph nodes indicated from the studies carried out with LYVE-1^-/-^ mice. Furthermore, in common with LYVE-1^-/-^ mice (Fig 5B), the reduction in bacterial burden in the draining lymph node of mice infected with M18 GAS was accompanied by a rise in bacterial burden in spleen (Fig 6C), and blood in LYVE-1 mAb pre-treated mice (Fig 6D). This effect was not seen in mice infected with M89 GAS and therefore may be specific to M18 GAS.

In order to better understand the influence of the M18 GAS LYVE-1 interaction on disease outcome and severity, we assessed the impact of LYVE-1 functional blockade on the induction of sepsis in mice following intra-muscular bacterial challenge over a 24 hour period (S6 Fig). Consistent with the results obtained at the 3 hour time point, LYVE-1 mAb blockade led to an almost ten-fold reduction in the number of bacteria recovered from the draining ipsilateral lymph node compared with IgG treated controls (S6 Fig). This was again coupled with an increase in bacterial dissemination into the blood circulation and spleen and, additionally at this time point, the liver (S6 Fig). In order to predict how this would impact on disease outcome, we measured the levels of serum IL-6, a sensitive marker of tissue inflammation. Although not significantly different, there was nevertheless a clear trend towards increased IL-6 levels in mice subjected to functional LYVE-1 blockade, (control IgG-treated mean: 1534, (SD: 372.9); LYVE-1 mAb-treated mean: 1848, (SD: 612.7)). Together these data provide evidence that LYVE-1 mediates interactions between the GAS capsule and lymphatic vessel endothelium that are critical for lymphatic tropism *in vivo*. Furthermore, in the case of hyper-encapsulated M18 GAS, this tropism may also limit systemic spread *via* the blood.

## Discussion

The host lymphatic network represents an ideal conduit for extracellular pathogens. Although recovery of GAS from draining lymph nodes in experimental disease and clinical syndromes are consistent with lymphatic spread [5], the underlying mechanisms for this potentially critical process and subsequent impact on disease outcome remain poorly characterized. In this investigation we demonstrate for the first time that the Gram positive extracellular pathogen, Group A streptococcus, can rapidly disseminate to draining lymph nodes *via* the lymphatic network, and that the process occurs through a specific interaction between the bacterial HA capsule and the lymphatic endothelial receptor, LYVE-1. We show by means of *in vitro* binding studies that expression of LYVE-1 is both necessary and sufficient to support adhesion of encapsulated GAS *in vitro*, and provide several lines of evidence indicating that the interaction between HA capsule and LYVE-1 is critical *in vivo* for GAS transit to draining lymph nodes. This was in stark contrast to isogenic acapsular GAS, which, although able to adhere to both human and murine LECs, did so in a non-specific and LYVE-1 independent manner and displayed a reduced ability to disseminate to draining lymph nodes *in vivo*.

Our studies have demonstrated a clear requirement for capsular HA in the interaction between GAS and LYVE-1. As documented for many pathogenic bacteria, GAS is known to vary capsule expression both *in vitro* and *in vivo*, a phenomenon known to be regulated by multiple factors including the key two-component regulatory system CovR/S [25,26]. In addition to this, capsule expression can be phenotypically fixed by genetic mutation, as exemplified by the hyper-encapsulated serotype M18, strains of which harbor a premature stop codon in the CovR/S regulator RocA [8], and acapsular serotypes M4 and M22, which completely lack the capsule operon [27]. The impact of serotype-specific variation in capsule expression on GAS adhesion to LYVE-1 and subsequent transit to draining lymph nodes is a subject that clearly warrants further study. While we report a similar reduction in transit of M18 and M89 GAS to draining lymph nodes following LYVE-1 receptor blockade, an accompanying increase in dissemination to the blood circulation and distant organs was only seen following M18 GAS infection, suggesting serotype specific variation in the role of the capsule LYVE-1 interaction during infection. We speculate that the interaction between M18 GAS and LYVE-1 augments association with lymphoid tissue and limits the spread to blood, thus providing a possible explanation for the infrequent association of M18 GAS with invasive disease and closer association with rheumatic fever [7].

It has previously been reported that mutations in the global GAS regulator CovR that normally represses capsule expression, can result in increased GAS dissemination to draining lymph nodes, a finding that can now be explained by the observations reported herein [28]. Previous data also demonstrate a role for the chemokine-cleaving protease SpyCEP in transit of GAS to draining lymph nodes, but the mechanism by which this occurs is still unknown [13]. As such the significance of lymphatic spread on the pathogenesis of individual GAS serotypes and other streptococcal species clearly warrants further investigation.

It is interesting to consider the extent to which lymphatic access *via* the LYVE-1:HA axis might contribute to the pathogenesis of other extracellular bacteria in addition to GAS. Several streptococcal species such as the zoonotic *S*. *zooepidemicus* and animal pathogens *S*. *equi* and *S*. *uberis* are known to express capsular HA [29]. Interestingly virulent *S*. *equi* displays a mucoid phenotype similar to that of hyper-encapsulated serotype M18 GAS, and is associated with the suppurative equine disease “strangles” that affects primarily the lymph nodes [30]. This further implicates the lymphatic system as a major conduit and focus for infection in domestic animals, possibly through an interaction with equine LYVE-1. Additional bacterial pathogens such as *Bacillus cereus* also express a capsule comprised of HA, while the closely related *B*. *anthracis* exhibits a single nucleotide polymorphism which results in a frame-shift that precludes the expression of HA [31]. One might speculate that such evolutionary loss of HA expression impairs access to the lymphatic network, resulting in increased transit through the blood circulation, as reported here, and may even be a critical determinant in the choice made by pathogens between these different vascular routes for establishing invasive disease. The observations made herein may well also be applicable to intracellular bacterial pathogens. It was recently reported that the Gram negative intracellular pathogen *Yersinia pestis* disseminates to draining lymph nodes, but that this process may not require an intracellular stage as previously thought [2]. These data suggest that the extracellular trafficking of pathogenic bacteria *via* the lymphatics may represent a universal mechanism for regulating systemic dissemination, in which LYVE-1 and other host receptors may play critical and previously unrecognized roles. In the case of GAS, the lymphatic tropism of heavily encapsulated bacteria, may act to restrict spread to the blood.

We have yet to determine exactly how LYVE-1:GAS interactions mediate lymphatic dissemination. The simplest explanation is that they promote streptococcal translocation into the lumen of lymphatic vessels and establishment of a reservoir in lymphoid tissue, much in the same way as GAS interactions with CD44 on the epithelial cell surface promote longevity of nasopharyngeal carriage and GAS persistence in the nasopharynx [10]. This hypothesis is supported by our observation that relatively few M18 GAS were recovered from lymph nodes draining the muscle infection site in LYVE-1^-/-^ mice compared with their wildtype counterparts. It is also notable that LYVE-1 is most abundant within the button-like junctions of initial lymphatics where migrating leukocytes enter from the surrounding tissues [32], and where the receptor may well facilitate their transit through hyaluronan-mediated interactions. Hence, it is tempting to speculate that HA encapsulated GAS hijack this normal immune mechanism to mediate their transit into lymphatics and then to draining lymph nodes. Whether some bacteria exit or-re-enter lymphatic vessels before reaching the lymph node is unknown; it seems possible that the clinical syndrome of lymphangitis could reflect either the presence of GAS in, or egress from, lymphatic vessels. GAS interaction with LYVE-1 may influence this and any subsequent inflammation.

The observed interaction between GAS and LYVE-1, and subsequent transit to draining lymph nodes can be regarded as benefitting either the pathogen or the host. It is widely accepted that the expression of capsular HA protects GAS against opsonization and phagocytosis by host immune cells, providing a mechanism by which encapsulated GAS could subvert any specialized innate response elicited by infected lymph nodes [33]. Results herein suggest that interaction with LYVE-1 or transit to lymph nodes, at least in the case of serotype M18 GAS, may prevent spread to the blood, insofar as blockade or genetic deletion of LYVE-1 not only prevented lymph node infection but promoted emergence in peripheral blood and distant organs. However, the interaction of GAS HA capsule with LYVE-1 may also provide an adhesive niche for the establishment of a persistent streptococcal reservoir within draining lymph nodes.

In conclusion, we describe a novel interaction between GAS capsular HA and the lymphatic endothelial receptor LYVE-1 that provides an important conduit by which encapsulated streptococci can disseminate into the lymphatic vasculature. To our knowledge this represents the first identification of a molecular mechanism for entry into the lymphatic system by an extracellular pathogen. Moreover, our findings provide a molecular explanation for the clinical observation that local lymphatic drainage plays a major role in limiting immediate systemic spread by GAS. The dissemination of extracellular bacteria in lymphatics may have far reaching relevance in the pathogenesis of both acute bacterial infection and post-infection sequelae.

## Methods

### Ethics statement

Normal donor cells were acquired from an approved sub collection of the Imperial College Tissue Bank. Use of anonymized human tissue was approved through the Oxford Regional Ethics Committee. *In vivo* experiments were performed in accordance with the Animals (Scientific Procedures) Act 1986, with appropriate UK Home Office licenses according to established institutional guidelines.

### GAS strains

Strains M18, M18_Δcapsule_ and M89 [8] were cultured on Columbia blood agar (CBA) or in Todd Hewitt broth (THB) at 37°C, 5% CO_2_. Strain M89_Δcapsule_ was constructed by electroporation with pUCMUT_hasKO_ (Table 1) as described previously [8].

**Table 1:** Bacterial strains used in this study.

Strain	Plasmid	Strain or gene alteration	Previously used
**M18**	-	Identical to H566	[8]
**M18_Δcapsule_**	pUCMUT_hasKO_	Capsule locus disruption mutant	[8]
**M89**	-	Identical to H395	[8]
**M89_Δcapsule_**	pUCMUT_hasKO_	Capsule locus disruption mutant	This study

### Cell culture and transfections

Primary HDLEC and MDLEC were derived from surgically resected human skin tissue and the skin of BALB/C pups respectively, using mAb MACS bead magnetic selection as described previously [22]. Purified cells were cultured in EGM-2 MV on gelatin-coated plastic (0.1%, Invitrogen). All cell lines were cultured at 37°C, 5% CO_2_ under humidified conditions and all experiments performed using confluent cell monolayers. Transfection of COS 7 with full-length human and mouse LYVE-1 cDNA and super-transfection of HDLEC with human LYVE-1 cDNA was achieved by lentiviral transduction.

### Lentiviral transduction of COS 7 and HDLEC with human and murine LYVE-1

Coding sequences of *lyve-1* were amplified from the appropriate cDNA using pfu Ultra AD (Agilent) with the following primers:

huLYVE-1–14 MluI F 5’ GCGACGCGTGAAGGGGTAGGCACGATGGCCAGG

huLYVE-1 969 * NotI R 5’ CGCGCGGCCGCCTAAACTTCAGCTTCCAGGCATCGCAC

mLYVE-1–14 MluI F 5’ GCGACGCGTGGAGGGATCTGCACAATGCTCCAG

mLYVE-1 957 * NotI R 5’ CGCGCGGCCGCCTAAAC TTCAGCTTCTAAGCATCGCAC

The amplified product was cloned into the lentiviral vector pHR Sin, based on the HIV retrovirus. 293T cells cultured in DMEM supplemented with 10% FCS were transiently transfected with pHR Sin plasmids together with pMD.G and p8.91 [34] using Genejuice (Merck, Darmstadt, Germany) according to the manufacturer’s instructions. Supernatant was harvested at 72hrs post-transfection and passed through a 0.45 micron filter to remove cell debris before transferring to COS 7 cells or HDLEC.

### Preparation of soluble recombinant human LYVE-1 ectodomains

The extracellular domain of human LYVE-1 (terminating after residue 238) in a 10 x histidine tag followed by a stop codon was amplified from a plasmid carrying full-length human LYVE-1 cDNA using the high fidelity polymerase pfu Ultra AD (Agilent). The construct was transfected into CHO cells and a clone with high expression levels was selected (kindly carried out by Janet Fennelly and Simon Davis; HIU, WIMM, Oxford). His-tagged protein was purified from culture supernatants using a 5 ml His Trap column (GE Healthcare). Following elution, the protein was buffer exchanged into PBS.

### Capsule quantification

GAS strains were cultured in THB and capsule quantified as described previously [8] using the hyaluronan DuoSet (R&D).

### Antibodies

Primary antibodies used were FITC-conjugated rabbit polyclonal anti-group A carbohydrate (Abcam ab68879. 0.2 mg/ml), goat polyclonal anti-human LYVE-1 (R&D AF2089), and goat polyclonal anti-mouse LYVE-1 (R&D AF2125). Blocking antibodies mAb20891 (R&D) and (BRIC235 IBGRL) were used against human LYVE-1 and CD44 respectively and mAb2125 (R&D) and ab25340 (KM201, Abcam) were used against mouse LYVE-1 and CD44 respectively. Blocking antibodies and isotype controls were used at 20 μg/ml.

### GAS adhesion assays

HDLECs, MDLECs and COS 7 cells were seeded at 5x10^5^ cells/well and once confluent, pre-treated with R10 media (RPMI, 10% FCS, L-glutamine) containing either control or blocking antibodies against LYVE-1 or CD44, high (>950 kDa) or ultralow (4–8 kDa) molecular weight HA (R&D Systems, UK), or recombinant soluble human LYVE-1 ectodomain for 20 minutes at room temperature. GAS were cultured on CBA and resuspended in PBS before being inoculated into each well at an MOI of 10. Adhesion assays were carried out following incubation for 30 minutes at 37°C, 5% CO_2_. Cells were washed 3 times with 1x PBS (Gibco) to remove non-adherent bacteria and cell-associated bacteria were quantified following lysis of the cell monolayer in sterile water. Replica wells of an 8-well chamber slide (Becton-Dickinson) inoculated with GAS pre-labelled with FITC-conjugated anti group A antibody (Abcam) were counter-stained with polyclonal anti-human or anti-mouse LYVE-1 antibodies as appropriate and imaged using a Zeiss Axiovert inverted epi-fluorescence microscope or a Zeiss LSM780 laser scanning inverted confocal microscope.

### Immunofluorescence antibody staining of mouse tissues

C57Bl/6 female mice (n = 3) were infected intra-dermally with 5×10^7^ M18 GAS and whole mount tissue sections and draining lymph nodes imaged 6 hours post infection. Whole mount tissue staining was carried out as described previously [2]. Frozen sections of lymph node were prepared by cryostat and fixed for 5 minutes in ice-cold acetone. Samples were stained with polyclonal anti-mouse LYVE-1 Ab and FITC-conjugated anti-group A carbohydrate antibody (Abcam). Whole mount tissue samples were viewed by Zeiss LSM780 confocal microscope, using either a Plan-Apochromat 10x/0.3DIC M27 (total magnification: 100x) or Plan-Apochromat 63x/1.4 oil (total magnification: 630x, resolution: 0.24 μm). For frozen lymph node sections, nuclei were counterstained with DAPI and sections were viewed on a Zeiss Axiovert S100 microscope equipped with epi-fluorescence.

### Murine intra-muscular infection

FVB/n female mice (4–5 weeks old (Charles River, Margate,UK)), C57Bl/6 and C57Bl/6 LYVE-1^-/-^ female mice (originally obtained from Regeneron Pharmaceuticals, USA) were challenged intra-muscularly with 1×10^8^ GAS, and quantitative endpoints compared at 3 or 24 hours post infection. For LYVE-1 blocking experiments mice were injected intra-peritoneally with 0.5 mg anti mLYVE-1 mAb2125 (R&D) or control rat IgG (isotype control or polyclonal) (R&D) 24 hours prior to infection. Mice were euthanized, blood taken by cardiac puncture and infected thigh muscle, spleen, liver, and left and right inguinal lymph nodes dissected. All organs were plated to quantify bacterial cfu and systemic dissemination. For imaging of draining inguinal lymph nodes, wildtype C57Bl/6 and C57Bl/6 LYVE-1^-/-^ mice (n = 3) were challenged intra-muscularly in both thighs with 1×10^8^ GAS. Three hours post infection lymph nodes were dissected and processed as described above for draining cervical lymph nodes, then stained with anti-mouse LYVE-1 (mAb C1/8), FITC-conjugated group A carbohydrate antibody (Abcam), anti-mouse podoplanin (eBioscience, clone eBio8.1.1) or anti-mouse PNAd (Biolegend, clone MECA79).

### Neutrophil phagocytosis assays

GAS pre-labelled with FITC-conjugated group A carbohydrate antibody (Abcam) were assayed for ability to resist uptake by purified human neutrophils as described previously [35].

### Statistics

All statistical analyses were performed with GraphPad Prism 5.0. Comparison of two datasets was carried out using unpaired Mann-Whitney and three or more data sets were analyzed by Kruskal-Wallis followed by Dunn's multiple comparison test. A p-value of ≤0.05 was considered significant.

## Supporting Information

S1 Fig Capsule enhances resistance of M18 but not M89 GAS to neutrophil-mediated uptake. Phagocytosis of M18 (A) and M89 (B) GAS and isogenic acapsular mutants quantified as percentage of neutrophils bearing intracellular GAS. (n = 3; Data represent mean+/-SD)
(TIF)

S2 Fig huLYVE-1 and mLYVE-1 are sufficient for binding of encapsulated GAS to COS 7 cells. (A) Lack of adhesion of M18 GAS (30 min incubation) to control untransfected COS 7 cells. (B) and (C): Binding of M18 GAS (30 min incubation) to COS 7 cells transfected with (B) huLYVE-1 or (C) mLYVE-1 in the presence of either control mAb (left) or LYVE-1 blocking mAb (right), demonstrated by immunofluorescence microscopy. Scale bars (20 μm) Red = LYVE-1, green = GAS, blue = nuclei
(TIF)

S3 Fig Blocking of CD44 does not impede adhesion of encapsulated GAS to HDLECs. Adhesion of M18 GAS to HDLECs. Left to right; quantitative culture (n = 3; Data represent mean+/-SD) and representative fluorescence microscopy of adherent GAS (30 min incubation) in the presence of control mAb or LYVE-1 blocking mAb. Scale bars (20 μm).
(TIF)

S4 Fig Acapsular GAS adhere to human and mouse derived LECs independently of LYVE-1. Adhesion of M18_Δcapsule_ GAS to HDLECs (A), LYVE-1 lentivirus-transfected HDLECs (B) and MDLECs (C). Left to right; quantitative culture (n = 4; Data represent mean+/-SD) and representative fluorescence microscopy of adherent GAS (30 min incubation) in the presence of control mAb or LYVE-1 blocking mAb. Scale bars (20 μm).
(TIF)

S5 Fig LYVE-1 functional blockade reduces M89 GAS dissemination to draining lymph nodes. Dissemination of M89 GAS in murine soft-tissue infection following LYVE-1 mAb blockade (n = 8/group). Numbers of GAS at site of infection (A) and ipsilateral lymph node (B), were determined by quantitative culture three hours post infection. Lines depict median values in each case.
(TIF)

S6 Fig Prolonged LYVE-1 functional blockade re-routes M18 GAS to the blood circulation. Dissemination of M18 GAS at 24h after onset of murine soft-tissue infection following LYVE-1 mAb blockade or control (n = 8/group). Numbers of GAS at site of infection (A), ipsilateral draining LN (B), spleen (C) blood (D) and liver (E) were determined by quantitative culture 24 hours post infection. Lines depict median values in each case (Mann Whitney U;* = p<0.05, ** = p<0.01).
(TIF)

## References

[1] Chtanova T, Schaeffer M, Han SJ, van Dooren GG, Nollmann M, Herzmark P, Chan SW, Satija H, Camfield K, Aaron H, Striepen B, Robey EA (2008) Dynamics of neutrophil migration in lymph nodes during infection. Immunity 29: 487–496. S1074–7613(08)00364–6 [pii]; 10.1016/j.immuni.2008.07.012 18718768 PMC2569002

[2] Gonzalez RJ, Lane MC, Wagner NJ, Weening EH, Miller VL (2015) Dissemination of a highly virulent pathogen: tracking the early events that define infection. PLoS Pathog 11: e1004587 10.1371/journal.ppat.1004587 PPATHOGENS-D-14–01893 [pii]. 25611317 PMC4303270

[3] Iannacone M, Moseman EA, Tonti E, Bosurgi L, Junt T, Henrickson SE, Whelan SP, Guidotti LG, von Andrian UH (2010) Subcapsular sinus macrophages prevent CNS invasion on peripheral infection with a neurotropic virus. Nature 465: 1079–1083. nature09118 [pii]; 10.1038/nature09118 20577213 PMC2892812

[4] Vindenes T, McQuillen D (2015) Images in clinical medicine. Acute lymphangitis. N Engl J Med 372:649 10.1056/NEJMicm1406569 25671257

[5] Bisno AL, Stevens DL (1996) Streptococcal infections of skin and soft tissues. N Engl J Med 334: 240–245. 10.1056/NEJM199601253340407 8532002

[6] Cole JN, Barnett TC, Nizet V, Walker MJ (2011) Molecular insight into invasive group A streptococcal disease. Nat Rev Microbiol 9: 724–736. nrmicro2648 [pii]; 10.1038/nrmicro2648 21921933

[7] Carapetis JR, Steer AC, Mulholland EK, Weber M (2005) The global burden of group A streptococcal diseases. Lancet Infect Dis 5: 685–694. S1473–3099(05)70267-X [pii]; 10.1016/S1473–3099(05)70267-X 16253886

[8] Lynskey NN, Goulding D, Gierula M, Turner CE, Dougan G, Edwards RJ, Sriskandan S (2013) RocA truncation underpins hyper-encapsulation, carriage longevity and transmissibility of serotype M18 group A streptococci. PLoS Pathog 9: e1003842 10.1371/journal.ppat.1003842 PPATHOGENS-D-13–01924 [pii]. 24367267 PMC3868526

[9] Wessels MR, Moses AE, Goldberg JB, DiCesare TJ (1991) Hyaluronic acid capsule is a virulence factor for mucoid group A streptococci. Proc Natl Acad Sci U S A 88: 8317–8321. 1656437 10.1073/pnas.88.19.8317 PMC52499

[10] Cywes C, Stamenkovic I, Wessels MR (2000) CD44 as a receptor for colonization of the pharynx by group A Streptococcus. J Clin Invest 106: 995–1002. 10.1172/JCI10195 11032859 PMC314343

[11] Schrager HM, Alberti S, Cywes C, Dougherty GJ, Wessels MR (1998) Hyaluronic acid capsule modulates M protein-mediated adherence and acts as a ligand for attachment of group A Streptococcus to CD44 on human keratinocytes. J Clin Invest 101: 1708–1716. 10.1172/JCI2121 9541502 PMC508753

[12] Cywes C, Wessels MR (2001) Group A Streptococcus tissue invasion by CD44-mediated cell signalling. Nature 414: 648–652. 10.1038/414648a 414648a [pii]. 11740562

[13] Kurupati P, Turner CE, Tziona I, Lawrenson RA, Alam FM, Nohadani M, Stamp GW, Zinkernagel AS, Nizet V, Edwards RJ, Sriskandan S (2010) Chemokine-cleaving Streptococcus pyogenes protease SpyCEP is necessary and sufficient for bacterial dissemination within soft tissues and the respiratory tract. Mol Microbiol 76: 1387–1397. MMI7065 [pii]; 10.1111/j.1365–2958.2010.07065.x 20158613 PMC2904501

[14] Loof TG, Rohde M, Chhatwal GS, Jung S, Medina E (2007) The contribution of dendritic cells to host defenses against Streptococcus pyogenes. J Infect Dis 196: 1794–1803. 10.1086/523647 18190260

[15] Harris EN, Weigel JA, Weigel PH (2004) Endocytic function, glycosaminoglycan specificity, and antibody sensitivity of the recombinant human 190-kDa hyaluronan receptor for endocytosis (HARE). J Biol Chem 279: 36201–36209. 10.1074/jbc.M405322200 M405322200 [pii]. 15208308

[16] Banerji S, Ni J, Wang SX, Clasper S, Su J, Tammi R, Jones M, Jackson DG (1999) LYVE-1, a new homologue of the CD44 glycoprotein, is a lymph-specific receptor for hyaluronan. J Cell Biol 144: 789–801. 10037799 10.1083/jcb.144.4.789 PMC2132933

[17] Jackson DG (2014) Lymphatic Regulation of Cellular Trafficking. J Clin Cell Immunol 5:258 10.4172/2155–9899.1000258 27808282 PMC5081098

[18] Caparon MG, Stephens DS, Olsen A, Scott JR (1991) Role of M protein in adherence of group A streptococci. Infect Immun 59: 1811–1817. 2019444 10.1128/iai.59.5.1811-1817.1991 PMC257920

[19] Courtney HS, Hasty DL, Dale JB (2002) Molecular mechanisms of adhesion, colonization, and invasion of group A streptococci. Ann Med 34: 77–87. 12108578 10.1080/07853890252953464

[20] Prevo R, Banerji S, Ferguson DJ, Clasper S, Jackson DG (2001) Mouse LYVE-1 is an endocytic receptor for hyaluronan in lymphatic endothelium. J Biol Chem 276: 19420–19430. 10.1074/jbc.M011004200 M011004200 [pii]. 11278811

[21] Nightingale TD, Frayne ME, Clasper S, Banerji S, Jackson DG (2009) A mechanism of sialylation functionally silences the hyaluronan receptor LYVE-1 in lymphatic endothelium. J Biol Chem 284: 3935–3945. M805105200 [pii]; 10.1074/jbc.M805105200 19033446

[22] Johnson LA, Clasper S, Holt AP, Lalor PF, Baban D, Jackson DG (2006) An inflammation-induced mechanism for leukocyte transmigration across lymphatic vessel endothelium. J Exp Med 203: 2763–2777. jem.20051759 [pii]; 10.1084/jem.20051759 17116732 PMC2118156

[23] Okada N, Liszewski MK, Atkinson JP, Caparon M (1995) Membrane cofactor protein (CD46) is a keratinocyte receptor for the M protein of the group A streptococcus. Proc Natl Acad Sci U S A 92: 2489–2493. 7708671 10.1073/pnas.92.7.2489 PMC42243

[24] Gale NW, Prevo R, Espinosa J, Ferguson DJ, Dominguez MG, Yancopoulos GD, Thurston G, Jackson DG (2007) Normal lymphatic development and function in mice deficient for the lymphatic hyaluronan receptor LYVE-1. Mol Cell Biol 27: 595–604. MCB.01503–06 [pii]; 10.1128/MCB.01503–06 17101772 PMC1800809

[25] Levin JC, Wessels MR (1998) Identification of csrR/csrS, a genetic locus that regulates hyaluronic acid capsule synthesis in group A Streptococcus. Mol Microbiol 30: 209–219. 9786197 10.1046/j.1365-2958.1998.01057.x

[26] Trevino J, Liu Z, Cao TN, Ramirez-Pena E, Sumby P (2013) RivR is a negative regulator of virulence factor expression in group A Streptococcus. Infect Immun 81: 364–372. IAI.00703–12 [pii]; 10.1128/IAI.00703–12 23147037 PMC3536152

[27] Flores AR, Jewell BE, Fittipaldi N, Beres SB, Musser JM (2012) Human disease isolates of serotype m4 and m22 group a streptococcus lack genes required for hyaluronic acid capsule biosynthesis. MBio 3: e00413–12. mBio.00413–12 [pii]; 10.1128/mBio.00413–12 23131832 PMC3487777

[28] Alam FM, Turner CE, Smith K, Wiles S, Sriskandan S (2013) Inactivation of the CovR/S virulence regulator impairs infection in an improved murine model of Streptococcus pyogenes naso-pharyngeal infection. PLoS One 8: e61655 10.1371/journal.pone.0061655 PONE-D-12–38382 [pii]. 23637876 PMC3636223

[29] Blank LM, Hugenholtz P, Nielsen LK (2008) Evolution of the hyaluronic acid synthesis (has) operon in Streptococcus zooepidemicus and other pathogenic streptococci. J Mol Evol 67: 13–22. 10.1007/s00239–008–9117–1 18551332

[30] Guss B, Flock M, Frykberg L, Waller AS, Robinson C, Smith KC, Flock JI (2009) Getting to grips with strangles: an effective multi-component recombinant vaccine for the protection of horses from Streptococcus equi infection. PLoS Pathog 5: e1000584 10.1371/journal.ppat.1000584 19763180 PMC2736577

[31] Oh SY, Budzik JM, Garufi G, Schneewind O (2011) Two capsular polysaccharides enable Bacillus cereus G9241 to cause anthrax-like disease. Mol Microbiol 80: 455–470. 10.1111/j.1365–2958.2011.07582.x 21371137 PMC3538873

[32] Baluk P, Fuxe J, Hashizume H, Romano T, Lashnits E, Butz S, Vestweber D, Corada M, Molendini C, Dejana E, McDonald DM (2007) Functionally specialized junctions between endothelial cells of lymphatic vessels. J Exp Med 204: 2349–2362. jem.20062596 [pii]; 10.1084/jem.20062596 17846148 PMC2118470

[33] Kastenmuller W, Torabi-Parizi P, Subramanian N, Lammermann T, Germain RN (2012) A spatially-organized multicellular innate immune response in lymph nodes limits systemic pathogen spread. Cell 150: 1235–1248. S0092–8674(12)00889–6 [pii]; 10.1016/j.cell.2012.07.021 22980983 PMC3514884

[34] Naldini L, Blomer U, Gallay P, Ory D, Mulligan R, Gage FH, Verma IM, Trono D (1996) In vivo gene delivery and stable transduction of nondividing cells by a lentiviral vector. Science 272: 263–267. 8602510 10.1126/science.272.5259.263

[35] Turner CE, Dryden M, Holden MT, Davies FJ, Lawrenson RA, Farzaneh L, Bentley SD, Efstratiou A, Sriskandan S (2013) Molecular analysis of an outbreak of lethal postpartum sepsis caused by Streptococcus pyogenes. J Clin Microbiol 51: 2089–2095. JCM.00679–13 [pii]; 10.1128/JCM.00679–13 23616448 PMC3697669

