# Supplementary figures and images for: Rapid Lymphatic Dissemination of Encapsulated Group A Streptococci via Lymphatic Vessel Endothelial Receptor-1 Interaction
Source: PLoS Pathog. 2015 Sep 9;11(9):e1005137. doi: 10.1371/journal.ppat.1005137 (PMC4564194; doi:10.1371/journal.ppat.1005137)

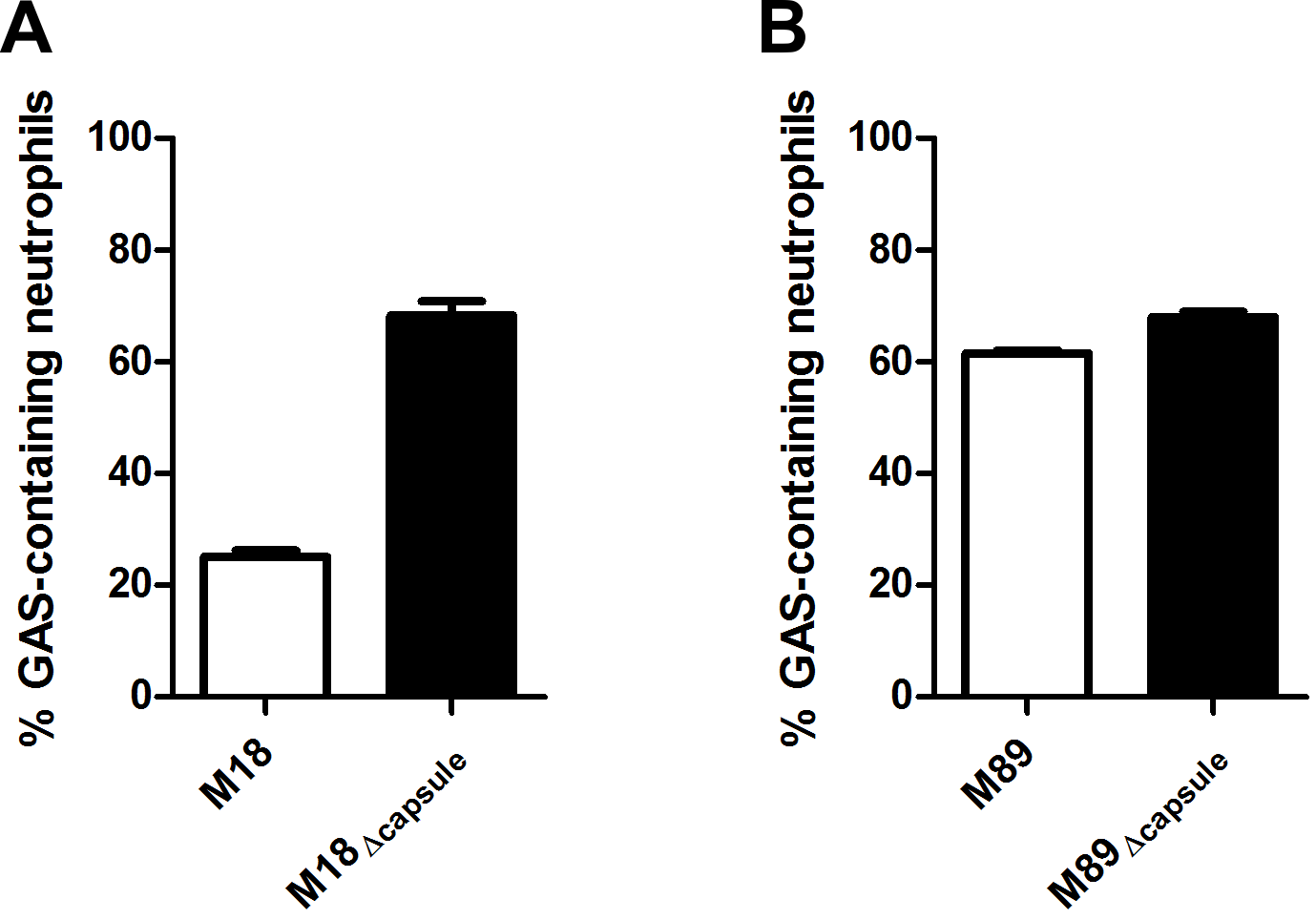

Supplement: S1 Fig — Phagocytosis of M18 (A) and M89 (B) GAS and isogenic acapsular mutants quantified as percentage of neutrophils bearing intracellular GAS. (n = 3; Data represent mean+/-SD) (TIF) [file ppat.1005137.s001.tif]

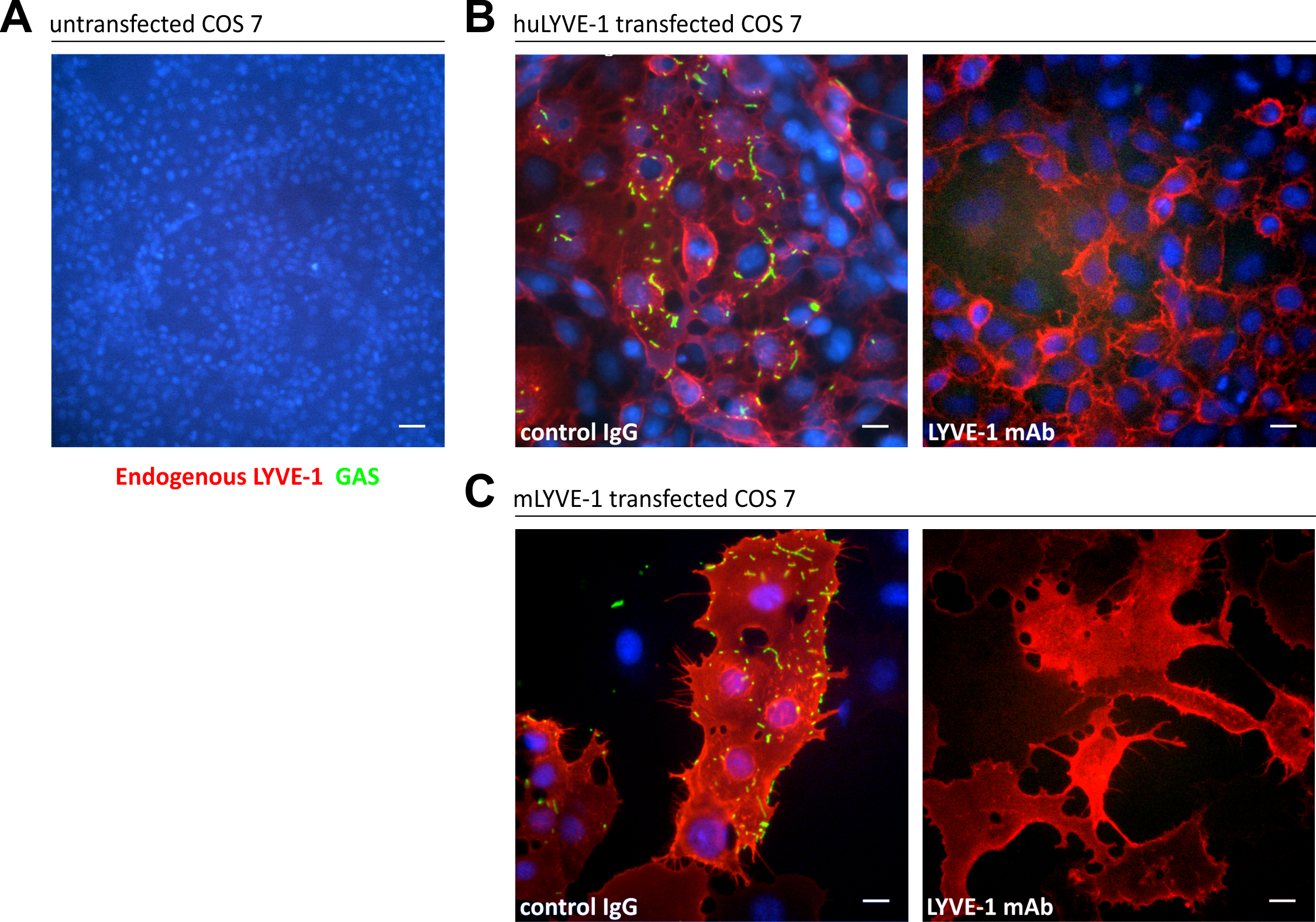

Supplement: S2 Fig — (A) Lack of adhesion of M18 GAS (30 min incubation) to control untransfected COS 7 cells. (B) and (C): Binding of M18 GAS (30 min incubation) to COS 7 cells transfected with (B) huLYVE-1 or (C) mLYVE-1 in the presence of either control mAb (left) or LYVE-1 blocking mAb (right), demonstrated by immunofluorescence microscopy. Scale bars (20 μm) Red = LYVE-1, green = GAS, blue = nuclei (TIF) [file ppat.1005137.s002.tif]

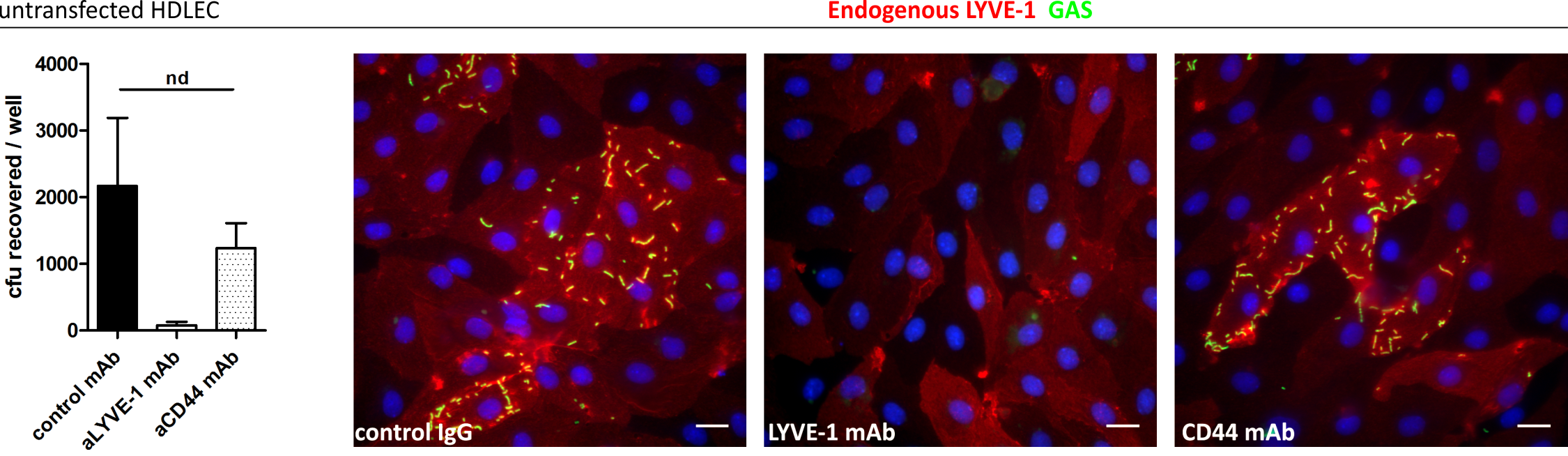

Supplement: S3 Fig — Adhesion of M18 GAS to HDLECs. Left to right; quantitative culture (n = 3; Data represent mean+/-SD) and representative fluorescence microscopy of adherent GAS (30 min incubation) in the presence of control mAb or LYVE-1 blocking mAb. Scale bars (20 μm). (TIF) [file ppat.1005137.s003.tif]

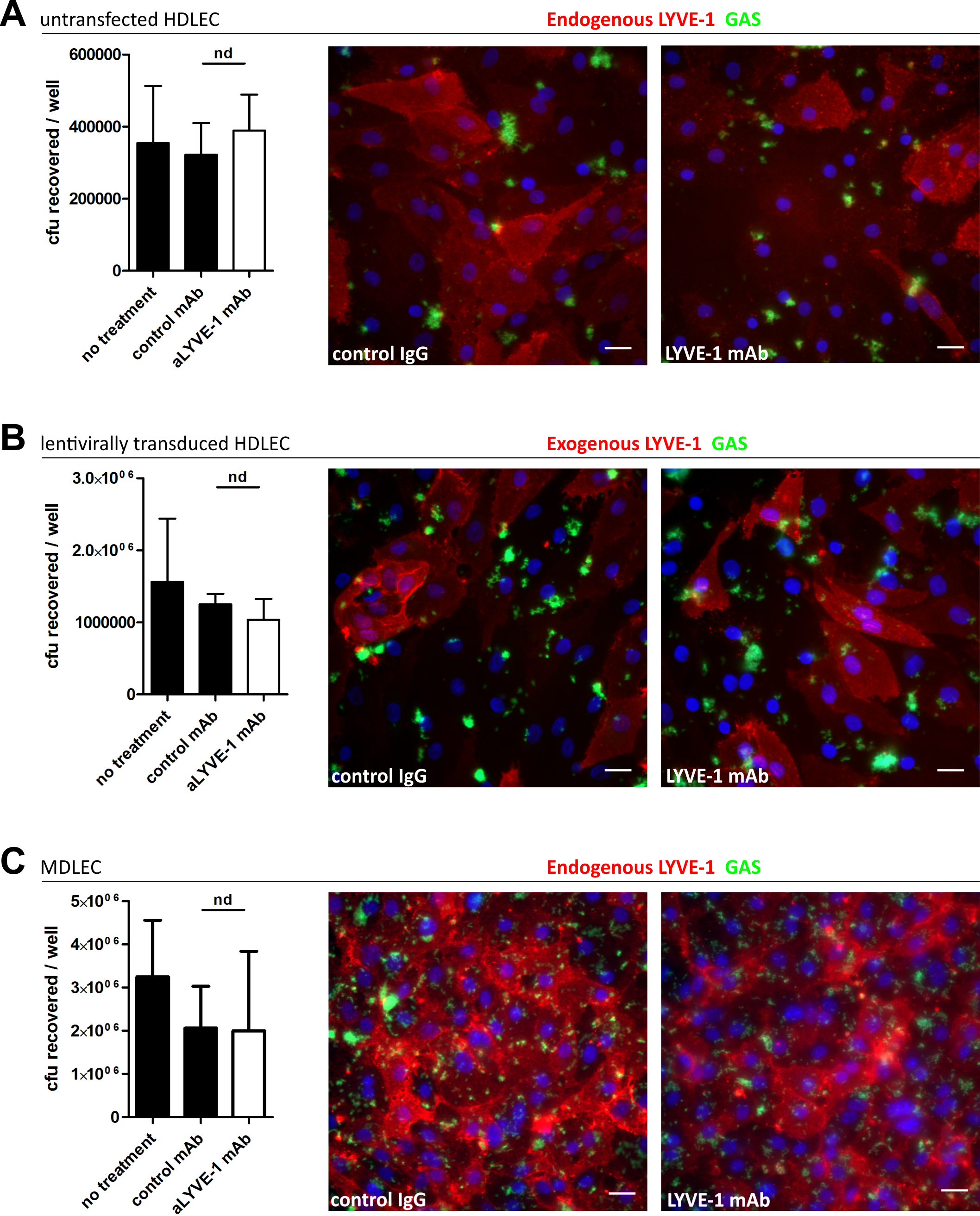

Supplement: S4 Fig — Adhesion of M18Δcapsule GAS to HDLECs (A), LYVE-1 lentivirus-transfected HDLECs (B) and MDLECs (C). Left to right; quantitative culture (n = 4; Data represent mean+/-SD) and representative fluorescence microscopy of adherent GAS (30 min incubation) in the presence of control mAb or LYVE-1 blocking mAb. Scale bars (20 μm). (TIF) [file ppat.1005137.s004.tif]

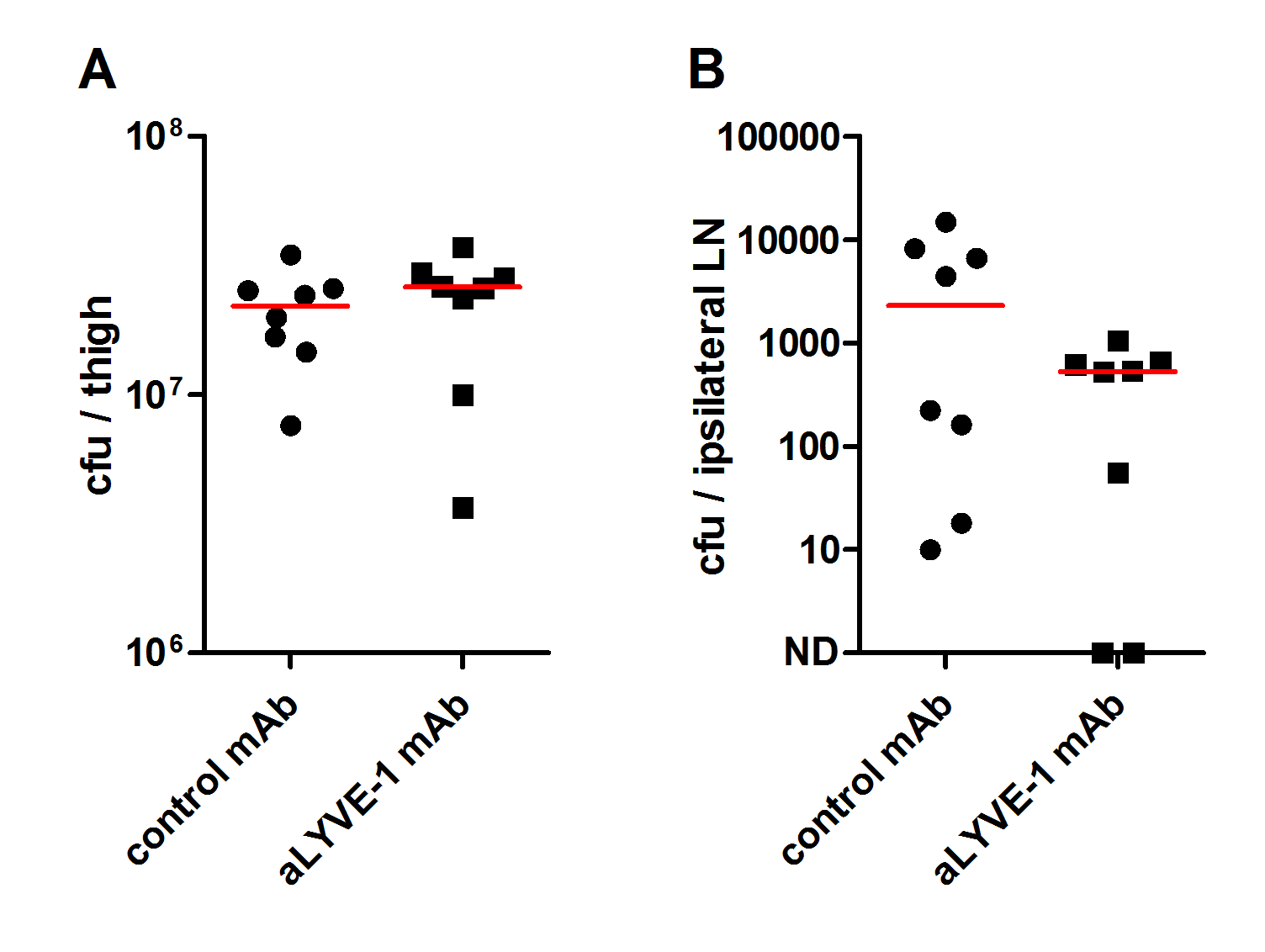

Supplement: S5 Fig — Dissemination of M89 GAS in murine soft-tissue infection following LYVE-1 mAb blockade (n = 8/group). Numbers of GAS at site of infection (A) and ipsilateral lymph node (B), were determined by quantitative culture three hours post infection. Lines depict median values in each case. (TIF) [file ppat.1005137.s005.tif]

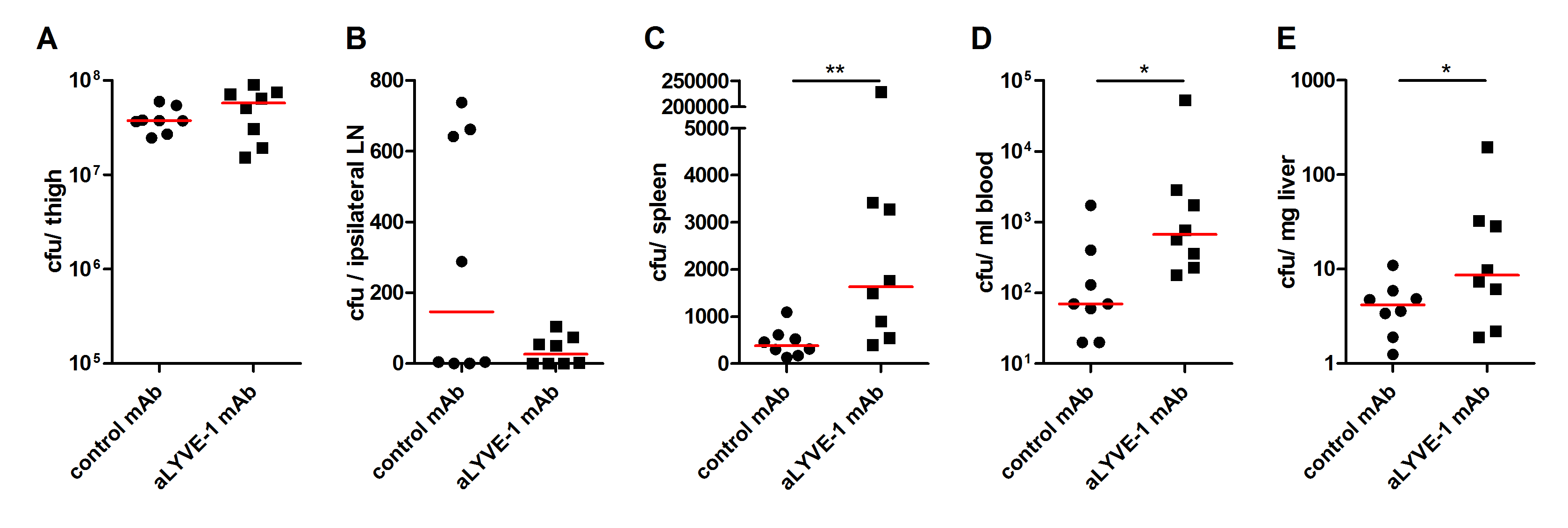

Supplement: S6 Fig — Dissemination of M18 GAS at 24h after onset of murine soft-tissue infection following LYVE-1 mAb blockade or control (n = 8/group). Numbers of GAS at site of infection (A), ipsilateral draining LN (B), spleen (C) blood (D) and liver (E) were determined by quantitative culture 24 hours post infection. Lines depict median values in each case (Mann Whitney U;* = p<0.05, ** = p<0.01). (TIF) [file ppat.1005137.s006.tif]
